# Non-Consent to a Wrist-Worn Accelerometer in Older Adults: The Role of Socio-Demographic, Behavioural and Health Factors

**DOI:** 10.1371/journal.pone.0110816

**Published:** 2014-10-24

**Authors:** Maliheh Hassani, Mika Kivimaki, Alexis Elbaz, Martin Shipley, Archana Singh-Manoux, Séverine Sabia

**Affiliations:** 1 Department of Epidemiology & Public Health, University College London, London, United Kingdom; 2 INSERM, U1018, Centre for Research in Epidemiology and Population Health, Villejuif, France; 3 University Paris 11, Villejuif, France; 4 University Versailles St-Quentin, Boulogne-Billancourt, France; 5 Centre de Gérontologie, Hôpital Ste Périne, AP-HP, Paris, France; Texas Tech University Health Science Centers, United States of America

## Abstract

**Background:**

Accelerometers, initially waist-worn but increasingly wrist-worn, are used to assess physical activity free from reporting-bias. However, its acceptability by study participants is unclear. Our objective is to assess factors associated with non-consent to a wrist-mounted accelerometer in older adults.

**Methods:**

Data are from 4880 Whitehall II study participants (1328 women, age range = 60–83), requested to wear a wrist-worn accelerometer 24 h every day for 9 days in 2012/13. Sociodemographic, behavioral, and health-related factors were assessed by questionnaire and weight, height, blood pressure, cognitive and motor function were measured during a clinical examination.

**Results:**

210 participants had contraindications and 388 (8.3%) of the remaining 4670 participants did not consent. Women, participants reporting less physical activity and less favorable general health were more likely not to consent. Among the clinical measures, cognitive impairment (Odds Ratio = 2.21, 95% confidence interval: 1.22–4.00) and slow walking speed (Odds Ratio = 1.38, 95% confidence interval: 1.02–1.86) were associated with higher odds of non-consent.

**Conclusions:**

The rate of non-consent in our study of older adults was low. However, key markers of poor health at older ages were associated with non-consent, suggesting some selection bias in the accelerometer data.

## Introduction

Physical activity is seen to be key to successful aging, [1] reducing risk of a range of chronic diseases [2] and cognitive [3] and physical [4] impairment. However, the ‘true’ effect of physical activity remains unclear as much of the research comes from studies where physical activity is self-reported, making associations subject to reporting biases. The correlation between objectively measured physical activity (e.g. accelerometry, doubly labelled water, heart rate monitor) and activity measured via questionnaire is typically low to moderate [5] and may be even lower in older adults. [6]–[9] Questionnaire-assessed measurements are prone to reporting bias, for example, due to social desirability or inaccurate recall, [5] and to measurement error since questionnaires include a limited number of items and do not capture the full range of physical activity undertaken over several days. [10] The low-to-moderate correlation between questionnaire and objective measures of physical activity, along with accelerometers becoming more affordable, is leading to an increasing use of accelerometers to measure physical activity, [11]–[20] including in studies on older adults. [11], [12], [20]

Accelerometry is a measure of one part of the body with inferences that apply to the whole body. It has the advantage of being free from reporting bias. In most previous studies, accelerometers are worn on the waist but moderate acceptance rate has led increasingly to the use of wrist-worn accelerometers. [21] However, its acceptability, especially among older persons, is unclear. [22], [23] Some studies have investigated the characteristics associated with non-wear time of waist-mounted accelerometers among those consenting to wear the device [24]–[26] but less is known about factors associated with non-consent. [26] Our objective was to assess non-consent to a wrist-worn accelerometer in older adults and examine the role of socio-demographic, behavioural, anthropometric, and health-related factors.

## Methods

### Study population

Data are drawn from the Whitehall II cohort study, established in 1985/88 on 10,308 individuals (67% men), aged 35–55 years. [27] Participants gave written consent to participate in the study and the University College London ethics committee approved the study. Study design consists of a clinical examination and a self-administered questionnaire. Since inception, socio-demographic, behavioural, and health-related factors, including self-reported physical activity via questionnaire, have been assessed approximately every five years (1985/88, 1991/93, 1997/99, 2002/04, 2007/09 and 2012/13).

### Accelerometer-assessed physical activity

Accelerometry was introduced the study at the 2012/13 clinical assessment for participants seen at the central London clinic and among those screened at home, living in the South-Eastern regions of England. A wrist-worn triaxial accelerometer (GeneActiv, Activinsights Ltd, Cambs, United Kingdom) was used, participants were asked to wear the waterproof accelerometer on their non-dominant wrist, non-stop for 9 consecutive (24-hour) days. They were also asked to complete a diary alongside wearing the accelerometer to report overnight sleep periods (falling asleep/standing up times), cycling and non-wear time. Among the 4880 participants who were offered the accelerometer, 388 did not consent and 210 had contraindications (allergies to metal or plastic (N = 40), travelling abroad (N = 168), other reasons (N = 2, strap too short and cognitive impairment)) and were not given the accelerometer.

### Socio-demographic factors

Demographic variables included in the analysis were age, sex, ethnicity (White, non-White) and marital status (married/cohabiting, other). Socioeconomic status (SES) measures included education and occupational position at 50 years. Education was defined as the highest qualification on leaving full-time education, categorized as university degree, higher secondary school, lower secondary school, and lower primary school or below. Occupational position was defined using the British civil service grade of employment and categorised as high (administrative), intermediate (professional or executive) and low (clerical or support grades). This measure in the Whitehall II data is a comprehensive marker of socioeconomic circumstances and is related to salary, social status, and level of responsibility at work. [27]

### Behavioural and anthropometric factors

*Smoking* was defined as current, ex-, and never smokers. *Alcohol consumption* was assessed via questions on the number of alcoholic drinks consumed in the last seven days, and categorized as “abstinence from alcohol” (no alcohol in the last week), “moderate alcohol consumption” (1–14 units/week in women, 1–21 units/week in men), and “heavy alcohol consumption” (≥15 units in women, ≥21 units in men). *Fruit and vegetable consumption* was assessed using the question “How often do you eat fresh fruit or vegetables?”; responses were on a 9-point scale, ranging from “seldom or never” to “3 or more times a day”. *Physical activity* was assessed using a 20-item questionnaire on frequency and duration of participation in different physical activities (e.g. walking, cycling, sports). Each activity was assigned a metabolic equivalent (MET) using a compendium of activity energy costs. [28] Duration of moderate and vigorous physical activity (≥3 MET) was used in the analysis. *Sedentary behavior* was assessed using two questions on sitting time: time spent sitting in front of a television (hours/week), and time sitting down for other activities (hours/week). Weight and height were assessed during the clinical examination and *body-mass index (BMI)* calculated as weight (in kilograms) divided by height (in meters) squared and categorized as follows: <25 kg/m^2^, 25–29.9 kg/m^2^, ≥30 kg/m^2^.

### Health-related characteristics

*Self-rated health* was assessed using the question “In general would you say your health is excellent, very good, good, fair, or poor?”.

*Blood pressure* was measured twice with the participant sitting after a 5-minute rest using the Hawksley random-zero sphygmomanometer. The average of two readings was taken to be the measured blood pressure. Hypertension was defined as systolic or diastolic blood pressure≥140 or ≥90 mm Hg respectively or use of antihypertensive drugs.

*Sleep-related characteristics* included measures of sleep duration and sleep disturbance. *Sleep duration* was assessed using the question “how many hours of sleep do you have on an average week-night?”.This variable was categorised as “≤5 hours”, “6–8 hours” and “≥9 hours”. *Sleep disturbance* was defined as self-report of having trouble falling asleep or staying asleep at least 21 nights per month. [29]

*Depressive symptoms* were assessed using the 20-item *Center for Epidemiologic Studies Depression (CES-D) scale*. Scores range between 0 and 60 with higher scores indicating greater depressive symptoms; scores ≥16 were used to represent cases of CES-D depression. [30], [31] Mental health was also measured using the *mental health component score of the short-form general health survey* (SF-36); higher scores indicate better mental health. [32] This variable was categorised into tertiles. The 30-item *Mini-Mental-State-Examination* (MMSE) was used to assess global cognitive status [33] and categorised as “normal” for scores ≥28, “slightly impaired” for scores between 24 and 27, and “impaired” for scores <24.

*Physical health* was assessed using questionnaires and clinical examination. The *physical component score of the SF-36* was used to measure self-rated physical health, and categorised into tertiles. [32] Higher scores represent better physical health. *Walking speed* was measured over a clearly marked 8-feet (2.44 m) walking course using a standardized protocol. [34], [35] Participants were asked to “walk to the other end of the course at [their] usual walking pace, just as if [they] were walking down the street to go the shops.” Three tests were performed and walking speed (m/s) was calculated as the distance divided by the mean of the three times to complete the test, and categorised into tertiles. Disability was assessed using questions on *basic (ADL)* [36] *and instrumental activities of daily living (IADL)*. [37] Participants reporting difficulties in one or more ADL or IADL were considered as having disability in ADL or IADL.

### Statistical analysis

Logistic regressions were used to assess the factors associated with non-consent to the accelerometer. We first assessed the association between socio-demographic variables with adjustment for age, sex, ethnicity, marital status, educational level and occupational position. Then, the association with lifestyle and anthropometric factors was investigated in a model adjusted for socio-demographic factors and mutually adjusted for smoking status, units of alcohol consumption, frequency of fruit and vegetable consumption, hours of moderate and vigorous physical activity, and BMI. Sitting time variables were added to the model separately as they had more missing values than the other covariates. Finally, the association with health-related factors was examined in models adjusted for socio-demographic variables and for each health-related variable separately due to potential collinearity. To test linearity in the association between these variables and non-consent, continuous (age, hours of moderate and vigorous physical activity, etc) and ordinal (educational level, self-rated general health, etc) variables were entered in the model as a linear term. In a final model, age and all variables associated with non-consent in previous analyses were entered in the logistic regression model simultaneously. The statistical analyses in this study were performed using SAS version 9.3 (SAS Institute, Inc., Cary, North Carolina) and STATA12 statistical software (StataCorp LP, College Station, Texas).

Sensitivity analyses were undertaken to examine the extent to which the associations with covariates tracked over time. In order to do this, we used covariates measured in 2002/04, that is 10 years before the assessment of accelerometer data, to examine associations with consenting to wear an accelerometer. For each covariate, missing values were replaced by data from the closest waves of data collection (1997/99 or 2007/09).

## Results

Among the 4880 participants offered the accelerometer (27.2% women, mean age  = 69.3 years, standard deviation (SD) = 5.7) at the clinical assessment, 210 presented contra-indications (allergies to metal or plastic, travelling abroad, strap too short, cognitive impairment). There were no sex differences (28.6% vs 27.2% women) but those with contra-indications were younger (68.3 vs 69.4 years), from higher occupational position (56.7% vs 44.3%) and higher educational level (42.9% vs 31.5% with university level, all P<0.05). Of the 4670 remaining participants, 388 (8.3%) did not consent to wear the accelerometer.

Among the socio-demographic factors investigated (Table 1), only being female was associated with higher non-consent (odds ratio (OR) = 1.74, 95% confidence interval (CI): 1.36–2.22). Of the behavioral factors (Table 2), only low levels of self-reported physical activity was associated with higher non-consent, the OR for non-consent per-hour lower reported moderate and vigorous activities = 1.04, 95%CI: 1.01–1.08; P for trend = 0.02.

**Table 1:** Association between socio-demographic factors and non-consent in the measure of physical activity by accelerometer.

Characteristics	N Non-consent/Total	% Non-consent	Non-consent OR† (95% CI)
Age (years)			
60–65	125/1461	8.6	1 (ref)
66–70	100/1396	7.2	0.84 (0.64, 1.11)
71–75	89/920	9.7	1.17 (0.87, 1.56)
76–83	74/893	8.3	0.99 (0.73, 1.35)
Sex			
Men	240/3402	7.1	1 (ref)
Women	148/1268	11.7	1.74 (1.36, 2.22)*
Ethnicity			
White	349/4303	8.1	1 (ref)
Non-white	39/367	10.6	1.14 (0.78, 1.67)
Marital status			
Married/cohabiting	282/3477	8.1	1 (ref)
Other	106/1193	8.9	0.92 (0.72, 1.19)
Educational level			
University degree	137/1473	9.3	1 (ref)
Higher secondary school	97/1293	7.5	0.79 (0.59, 1.05)
Lower secondary school	113/1462	7.7	0.77 (0.57, 1.04)
Primary school or below	41/442	9.3	0.81 (0.53, 1.25)
Occupational position at 50y			
High	171/2070	8.3	1 (ref)
Intermediate	155/2066	7.5	0.91 (0.70, 1.18)
Low	62/534	11.6	1.15 (0.76, 1.74)

*P<0.05.

† Odds ratios are mutually adjusted for all socio-demographic factors listed in the table (N = 4670).

**Table 2:** Association of behavioural and anthropometric factors with non-consent to the measure of physical activity by accelerometer.

Characteristics	N Non-consent/Total	% Non-consent	Non-consent OR (95% CI)
Smoking status†			
Never smokers	218/2443	8.9	1 (ref)
Ex-smokers	148/2001	7.4	0.87 (0.70, 1.10)
Current smokers	14/146	9.6	1.07 (0.60, 1.92)
Alcohol consumption in the previous week†			
None	85/882	9.6	1.10 (0.83, 1.45)
Moderate	241/3023	8.0	1 (ref)
Heavy	54/685	7.9	1.02 (0.75, 1.40)
Fruit and vegetable consumption†			
Twice daily	220/2685	8.2	1 (ref)
Daily	73/959	7.6	0.99 (0.75, 1.32)
Less than daily	87/946	9.2	1.21 (0.92, 1.59)
Moderate and vigorous physical activity†			
>4 hours/week	111/1591	7.0	1 (ref)
1–4 hours/week	144/1688	8.5	1.14 (0.88, 1.48)
≤1 hours/week	125/1311	9.5	1.23 (0.92, 1.63)
*Per 1 hour/week decrement*			*1.04 (1.01, 1.08)* *
Sitting down watching TV‡			
<14 hours/week	100/1112	9.0	1 (ref)
14–23 hours/week	138/1730	8.0	0.91 (0.69, 1.20)
≥23 hours/week	130/1621	8.0	0.89 (0.66, 1.20)
Sitting down for other activities‡			
<17 hours/week	126/1433	8.8	1 (ref)
17–26 hours/week	84/1135	7.4	0.83 (0.62, 1.11)
≥26 hours/week	158/1895	8.3	0.93 (0.72, 1.20)
BMI†			
<25 kg/m^2^	142/1751	8.1	1 (ref)
25–29.9 kg/m^2^	159/1994	8.0	1.03 (0.81, 1.31)
≥30 kg/m^2^	79/845	9.4	1.11 (0.82, 1.49)

*P<0.05.

† Odds ratios are adjusted for age, sex, ethnicity, marital status, educational level, and occupational position at 50y, smoking status, alcohol consumption, fruit and vegetable consumption, hours of moderate and vigorous physical activity and BMI (N = 4590).

‡ Analyses are additionally adjusted for the sitting variables and based on a smaller sample due to a higher number of missing values for these variables (N = 4463).

Table 3 shows that non-consent was higher in participants reporting good to poor general health compared to excellent/very good health, in those with hypertension (OR = 1.26, 95%CI: 1.02–1.57), sleep duration ≤5 hours/night (OR = 1.40, 95%CI: 1.00–1.96), MMSE score≤23 (OR = 2.25, 95%CI: 1.23–4.11), and slower walking speed (OR = 1.58, 95%CI: 1.18–2.12 for lower vs higher tertile, P for trend = 0.03). In analyses (Table 4) adjusted for age and all covariates associated with non-consent (Tables 1–3), associations remained evident for sex, duration of moderate-vigorous physical activity, self-reported general health, MMSE score and walking speed (P<0.05), but not hypertension and sleep duration.

**Table 3:** Association of health-related factors with non-consent to the measure of physical activity by accelerometer†.

Characteristics	N Non-consent/Total	% Non-consent	Non-consent OR† (95% CI)
Self-reported general health			
Excellent/very good	153/2211	6.9	1 (ref)
Good	179/1852	9.7	1.42 (1.13, 1.79)*
Fair to poor	53/514	9.4	1.33 (0.95, 1.87)
Hypertension			
No	166/2216	7.5	1 (ref)
Yes	222/2454	9.1	1.26 (1.02, 1.57)*
Difficulty falling asleep or staying asleep			
<20 days/month	336/4197	8.0	1 (ref)
≥21 days/month	43/398	10.8	1.38 (0.98, 1.94)
Sleep duration			
≤5 hours	44/389	11.3	1.40 (1.00, 1.96)
6–8 hours	325/4112	7.9	1 (ref)
≥9 hours	12/127	9.5	1.24 (0.67, 2.27)
CESD score			
0–15	326/4063	8.0	1 (ref)
≥16	54/539	10.0	1.16 (0.85, 1.59)
SF36 mental component score			
Higher tertile: ≥58.1	114/1544	7.4	1 (ref)
Second tertile: 53.4–58.1	122/1540	7.9	1.07 (0.82, 1.40)
Lower tertile: <53.4	145/1554	9.3	1.24 (0.95, 1.60)
SF36 physical component score			
Higher tertile: ≥53.4	126/1543	8.2	1 (ref)
Second tertile: 47.3–53.4	120/1545	7.8	0.93 (0.72, 1.22)
Lower tertile: <47.3	135/1550	8.7	0.99 (0.76, 1.29)
Cognitive status			
Normal (MMSE≥28)	260/3419	7.6	1 (ref)
Slightly impaired (MMSE 24–27)	98/1097	8.9	1.20 (0.93, 1.56)
Impaired (MMSE≤23)	16/94	17.0	2.25 (1.23, 4.11)*
Walking speed			
Higher tertile: ≥1.26 m/s	97/1534	6.3	1 (ref)
Second tertile: 1.04–1.25 m/s	114/1523	7.5	1.15 (0.86, 1.53)
Lower tertile: <1.04 m/s	157/1547	10.2	1.58 (1.18, 2.12)*
ADL disability			
None	347/4170	8.3	1 (ref)
≥1	38/451	8.4	1.01 (0.71, 1.44)
IADL disability			
None	313/3770	8.3	1 (ref)
≥1	71/849	8.4	0.93 (0.70, 1.22)

*P<0.05.

† Each variable was entered separately in a model adjusted for age, sex, ethnicity, marital status, educational level, and occupational position at 50y. N varied from one analysis to another due to missing values in the variable of interest (N varied between 4595 and 4670).

**Table 4:** Fully adjusted model of factors associated with non-consent in the measure of physical activity by accelerometer.

Characteristics	Non-consent OR† (95% CI)
Age (years)	
60–65	1 (ref)
66–70	0.77 (0.58, 1.02)
71–75	0.96 (0.71, 1.31)
76–83	0.72 (0.51, 1.01)
Sex	
Men	1 (ref)
Women	1.39 (1.09, 1.77)*
Moderate and vigorous physical activity	
Per 1 hour/week decrement	1.04 (1.00, 1.08)*
Self-reported general health	
Excellent/very good	1 (ref)
Good	1.27 (1.00, 1.62)*
Fair to poor	0.96 (0.66, 1.40)
Hypertension	
No	1 (ref)
Yes	1.11 (0.88, 1.39)
Sleep duration	
≤5 hours	1.14 (0.79, 1.64)
6–8 hours	1 (ref)
≥9 hours	1.12 (0.59, 2.12)
Cognitive status	
Normal (MMSE≥28)	1 (ref)
Slightly impaired (MMSE 24–27)	1.12 (0.87, 1.45)
Impaired (MMSE≤23)	2.21 (1.22, 4.00)*
Walking speed	
Higher tertile: ≥1.26 m/s	1 (ref)
Second tertile: 1.04–1.25 m/s	1.11 (0.83, 1.48)
Lower tertile: <1.04 m/s	1.38 (1.02, 1.86)*

*P<0.05.

† Odds ratios are mutually adjusted (N = 4496).

Sensitivity analyses using covariates from 2002/04 showed similar results as those from the main analysis. In addition, overweight (OR = 1.24, 95%CI: 0.98; 1.58) and lower mental component scores (OR for 1^st^ vs 3^rd^ tertile = 1.33, 95%CI = 1.02, 1.74) were also associated with higher odds of non-consent (Tables S1–S3 in File S1). However, in the fully adjusted model (Table S4 in File S1), these associations were no longer apparent and as with covariates assessed in 2012/13, higher odds of non-consent were found in women, participants reporting lower physical activity, and those with slower walking speed. Only 13 participants classified as being cognitively impaired in 2002/04, thus reducing the power to detect an association.

## Discussion

In a British cohort of 4880 older adults aged 60 to 83 years, only 8.3% of eligible participants did not consent to wear a wrist-worn accelerometer. Women, participants reporting lower physical activity, less favorable general health, those with cognitive impairment and slower walking speed were more likely not to consent. The associations with sex, physical activity, and motor function were also evident when these factors were assessed 10 years before the accelerometer, showing that correlates of non-consent track over time.

A recent trend towards more comprehensive assessment of physical activity has been observed with accelerometers increasingly used in research settings. [11]–[20] Unlike physical activity questionnaires, accelerometers have the advantage of not being affected by reporting bias. However, previous studies reported a moderate acceptance rate for waist-worn accelerometers, potentially leading to selection bias in subsequent analysis. In the 2003-2006 National Health and Nutrition Examination Survey (NHANES), the response rate was 68%. [18] In the on-going physical activity assessment of physical activity in Women Health Study, it was around 63%. [20] In our study, using a wrist rather than a waist-worn accelerometer, the response rate at 92% was much higher in participants without contraindications. Previous studies that have used a wrist-worn accelerometer in older adults (on average 80 years) reported similar acceptance rates (around 90%). [22], [23] The NHANES moved from a hip- to a wrist-worn accelerometer between 2003–2006 and 2011–2012 surveys; preliminary results suggest improved compliance rates, from 40 to 70% (varying by age group) to 70 to 80% participants providing data over six or more days. [21] Thus, the wrist-worn accelerometer appears to be better accepted then waist-worn devices.

Few previous studies have investigated factors associated with non-response, and much of the evidence is still on waist-worn devices. [24]–[26] Furthermore, the focus has been on factors associated with non-wear time, [24], [25] as this is a problem with waist-worn devices. Our use of a wrist-worn accelerometer shows that only a small fraction (72 (1.5%) participants) did not wear the accelerometer for a length of time deemed to be sufficient (at least 16 hours on 2 week-end days and 2 weekdays, criteria for data validity [38]). However, the present study highlights the issue on non-consent among participants who attended the medical assessments, and were thus clearly “responders” in our longitudinal study.

In a substudy of the Health Survey of England (N = 2263, mean age = 52y, SD = 18), no difference in socio-demographic, anthropometric, behavioural, and health-related factors were observed between the 1724 individuals who consented to wear a waist-worn accelerometer and provided sufficient data (≥4 days with ≥10 hours of wear time) and 302 (13%) participants who declined to wear the accelerometer. [26] However, difference in wear-time were observed by age and smoking status. [26] Among the 2003–2004 NHANES participants who provided accelerometer data, older participants, non-Hispanic White, those with higher education, married, non-current smokers, and those with a better health profile were more likely to wear the accelerometer for at least 4 days for at least 10 hours. [25] In the present study, women, participants reporting less physical activity and less favourable general health, those cognitively impaired and those with slower walking speed were under-represented compared to the target population which points to a potential source of selection bias. For example, studies on the association between physical activity and cognitive or motor function might be biased due to greater non-consent in some groups. Overall, these results along with those presented in previous studies suggest that the factors associated with non-participation and non-wear time differ by position of wear of the accelerometer and study population, highlighting the importance of identifying these factors in future studies.

Our study has several strengths including its large study sample, the use of a waterproof wrist-worn accelerometer, and a range of covariates included in the analyses. It also has limitations. Apart from BMI, hypertension, global cognitive status and walking speed, most covariates were self-reported. Furthermore, although the sample covered a wide socioeconomic range, data are from an occupational cohort and cannot be considered to be representative of the general population. Finally, the low rate of non-participation is a strength for the study but it is a limitation for the present analysis since some associations might not have been detected due to low power.

In summary, among the participants from the British Whitehall II cohort study aged 60 to 83 years, the rate of non-consent to wear a wrist-worn accelerometer was low (8.3%). Sex, physical activity level, self-rated general health, cognitive and motor function were associated with non-consent. Our findings suggest that although wrist-worn accelerometers have lower rates of non-wear time, the extracted data is subject to some selection bias due to higher non-consent in some groups. Future studies are required to examine the generalisability of our across different populations.

## Supporting Information

File S1 **Tables S1–S4.** Table S1. Association between socio-demographic factors in 2002/04 and non-consent in the measure of physical activity by accelerometer in 2012/13. Table S2. Association of behavioural and anthropometric factors in 2002/04 with non-consent to the measure of physical activity by accelerometer in 2012/13. Table S3. Association of health-related factors with non-consent to the measure of physical activity by accelerometer^†^. Table S4. Fully adjusted model of factors associated with non-consent in the measure of physical activity by accelerometer.
(DOCX)
