# Supplementary material for: Non-Consent to a Wrist-Worn Accelerometer in Older Adults: The Role of Socio-Demographic, Behavioural and Health Factors
Source: PLoS One. 2014 Oct 24;9(10):e110816. doi: 10.1371/journal.pone.0110816 (PMC4208789; doi:10.1371/journal.pone.0110816)
Supplement: File S1 — Tables S1–S4. Table S1. Association between socio-demographic factors in 2002/04 and non-consent in the measure of physical activity by accelerometer in 2012/13. Table S2. Association of behavioural and anthropometric factors in 2002/04 with non-consent to the measure of physical activity by accelerometer in 2012/13. Table S3. Association of health-related factors with non-consent to the measure of physical activity by accelerometer†. Table S4. Fully adjusted model of factors associated with non-consent in the measure of physical activity by accelerometer. (DOCX) [file pone.0110816.s001.docx]

Table S1. Association between socio-demographic factors in 2002/04 and non-consent in the measure of physical activity by accelerometer in 2012/13

| **Characteristics** | **N**  **Non-consent /Total** | **%**  **Non-consent** | **Non-consent**  **OR**^†^ **(95% CI)** |
| --- | --- | --- | --- |
| Age (years) |  |  |  |
| 50-55 | 130/1480 | 8.8 | 1 (ref) |
| 56-50 | 98/1362 | 7.2 | 0.82 (0.62, 1.08) |
| 51-55 | 86/911 | 9.4 | 1.10 (0.82, 1.47) |
| 56-83 | 74/917 | 8.1 | 0.94 (0.69, 1.28) |
| Sex |  |  |  |
| Men | 240/3402 | 7.1 | 1 (ref) |
| Women | 148/1268 | 11.7 | 1.77 (1.39, 2.26)* |
| Ethnicity |  |  |  |
| White | 349/4303 | 8.1 | 1 (ref) |
| Non-white | 39/367 | 10.6 | 1.19 (0.82, 1.75) |
| Marital status |  |  |  |
| Married/cohabiting | 291/3568 | 8.2 | 1 (ref) |
| Other | 97/1102 | 8.8 | 0.93 (0.72, 1.20) |
| Educational level |  |  |  |
| University degree | 137/1473 | 9.3 | 1 (ref) |
| Higher secondary school | 97/1293 | 7.5 | 0.79 (0.60, 1.05) |
| Lower secondary school | 113/1462 | 7.7 | 0.79 (0.59, 1.05) |
| Primary school or below | 41/442 | 9.3 | 0.86 (0.56, 1.32) |
| Occupational position |  |  |  |
| High | 183/2235 | 8.2 | 1 (ref) |
| Intermediate | 156/1986 | 7.9 | 0.91 (0.72, 1.20) |
| Low | 49/449 | 10.9 | 1.02 (0.67, 1.57) |

*P<0.05

^†^Odds ratios are mutually adjusted for all socio-demographic factors listed in the table (N=4670).

Table S2. Association of behavioural and anthropometric factors in 2002/04 with non-consent to the measure of physical activity by accelerometer in 2012/13

| **Characteristics** | | **N**  **Non-consent /Total** | **%**  **Non-consent** | **Non-consent**  **OR (95% CI)** |
| --- | --- | --- | --- | --- |
| Smoking status^†^ | |  |  |  |
| Never smokers | | 206/2317 | 8.8 | 1 (ref) |
| Ex-smokers | | 143/1945 | 7.4 | 0.88 (0.70, 1.11) |
| Current smokers | | 28/317 | 8.9 | 1.01 (0.66, 1.54) |
| Alcohol consumption in the previous week^†^ | |  |  |  |
| None | | 70/638 | 9.9 | 1.09 (0.81, 1.48) |
| Moderate | | 232/2691 | 7.9 | 1 (ref) |
| Heavy | | 75/873 | 7.9 | 1.03 (0.78, 1.37) |
| Fruit and vegetable consumption^†^ | |  |  |  |
| Twice daily | | 149/1883 | 7.9 | 1 (ref) |
| Daily | | 143/1624 | 8.8 | 1.17 (0.91, 1.50) |
| Less than daily | | 85/987 | 7.9 | 1.06 (0.79, 1.43) |
| Moderate and vigorous physical activity^†^ |  | |  |  |
| >4 hours/week | | 123/1837 | 6.7 | 1 (ref) |
| 1-4 hours/week | | 148/1728 | 8.6 | 1.22 (0.94, 1.57) |
| ≤ 1 hours/week | | 106/1014 | 10.5 | 1.48 (1.11, 1.97)* |
| *Per 1 hour/week decrement* | |  |  | *1.05 (1.01, 1.09)** |
| BMI^†^ | |  |  |  |
| <25 kg/m² | | 131/1717 | 7.6 | 1 (ref) |
| 25-29.9 kg/m² | | 182/2085 | 8.7 | 1.24 (0.98, 1.58) |
| ≥30 kg/m² | | 64/777 | 8.2 | 1.02 (0.74, 1.40) |

*P<0.05

^†^ Odds ratios are adjusted for age, sex, ethnicity, marital status, educational level, and occupational position at 50y, smoking status, alcohol consumption, fruit and vegetable consumption, hours of moderate and vigorous physical activity and BMI (N=4579).

Table S3. Association of health-related factors with non-consent to the measure of physical activity by accelerometer^†^

| **Characteristics** | **N**  **Non-consent /Total** | **%**  **Non-consent** | **Non-consent**  **OR**^†^ **(95% CI)** |
| --- | --- | --- | --- |
| Self-reported general health |  |  |  |
| Excellent/very good | 173/2354 | 7.4 | 1 (ref) |
| Good | 145/1651 | 8.8 | 1.19 (0.94, 1.51) |
| Fair to poor | 63/602 | 10.5 | 1.38 (1.01, 1.89)* |
| Hypertension |  |  |  |
| No | 232/3011 | 7.7 | 1 (ref) |
| Yes | 150/1613 | 9.3 | 1.22 (0.98, 1.52) |
| Difficulty falling asleep or staying asleep |  |  |  |
| < 20 days/month | 349/4277 | 8.2 | 1 (ref) |
| ≥ 21 days/month | 32/323 | 9.9 | 1.17 (0.79, 1.71) |
| Sleep duration |  |  |  |
| ≤5 hours | 33/359 | 9.2 | 1.11 (0.76, 1.62) |
| 6-8 hours | 339/4155 | 8.2 | 1 (ref) |
| ≥9 hours | 9/88 | 10.2 | 1.33 (0.66, 2.70) |
| CESD score |  |  |  |
| 0-15 | 314/3917 | 8.0 | 1 (ref) |
| ≥ 16 | 60/642 | 9.4 | 1.11 (0.82, 1.50) |
| SF36 mental component score |  |  |  |
| Higher tertile: ≥ 57.1 | 112/1532 | 7.3 | 1 (ref) |
| Second tertile: 51.5-57.1 | 120/1523 | 7.9 | 1.09 (0.83, 1.43) |
| Lower tertile: < 51.5 | 148/1523 | 9.7 | 1.33 (1.02, 1.74)* |
| SF36 physical component score |  |  |  |
| Higher tertile: ≥ 54.0 | 132/1534 | 8.6 | 1 (ref) |
| Second tertile: 48.8-54.0 | 112/1510 | 7.4 | 0.84 (0.64, 1.09) |
| Lower tertile: < 48.8 | 136/1534 | 8.9 | 0.95 (0.73, 1.23) |
| Cognitive status |  |  |  |
| Normal (MMSE≥28) | 325/3986 | 7.6 | 1 (ref) |
| Slightly impaired (MMSE 24-27) | 47/578 | 8.9 | 0.92 (0.66, 1.29) |
| Impaired (MMSE≤23) | 2/13 | 17.0 | 1.51 (0.32, 7.08) |
| Walking speed |  |  |  |
| Higher tertile: ≥ 1.36 m/s | 97/1534 | 6.3 | 1 (ref) |
| Second tertile: 1.15-1.36 m/s | 132/1512 | 8.7 | 1.40 (1.06, 1.84)* |
| Lower tertile: <1.15 m/s | 148/1529 | 9.7 | 1.39 (1.04, 1.85)* |

*P<0.05

^†^ Each variable was entered separately in a model adjusted for age, sex, ethnicity, marital status, educational level, and occupational position at 50y. N varied from one analysis to another due to missing values in the variable of interest (N varied between 4559 and 4624).

Table S4. Fully adjusted model of factors associated with non-consent in the measure of physical activity by accelerometer

| **Characteristics** | **Non-consent**  **OR**^†^ **(95% CI)** |
| --- | --- |
| Age (years) |  |
| 50-55 | 1 (ref) |
| 56-50 | 0.83 (0.62, 1.10) |
| 51-55 | 1.11 (0.82, 1.51) |
| 56-83 | 0.94 (0.68, 1.29) |
| Sex |  |
| Men | 1 (ref) |
| Women | 1.63 (1.29, 2.06)* |
| Moderate and vigorous physical activity |  |
| Per 1 hour/week decrement | 1.04 (1.00, 1.08)* |
| BMI |  |
| <25 kg/m² | 1 (ref) |
| 25-29.9 kg/m² | 1.15 (0.90, 1.46) |
| ≥30 kg/m² | 0.91 (0.65, 1.26) |
| Self-reported general health |  |
| Excellent/very good | 1 (ref) |
| Good | 1.06 (0.83, 1.35) |
| Fair to poor | 1.15 (0.82, 1.61) |
| Hypertension |  |
| No | 1 (ref) |
| Yes | 1.26 (1.00, 1.58) |
| SF36 mental component score |  |
| Higher tertile: ≥ 57.1 | 1 (ref) |
| Second tertile: 51.5-57.1 | 1.07 (0.81, 1.41) |
| Lower tertile: < 51.5 | 1.23 (0.93, 1.63) |
| Walking speed |  |
| Higher tertile: ≥ 1.26 m/s | 1 (ref) |
| Second tertile: 1.04-1.25 m/s | 1.37 (1.04, 1.81)* |
| Lower tertile: <1.04 m/s | 1.28 (0.96, 1.70) |

*P<0.05

^†^ Odds ratios are mutually adjusted (N=4525).
